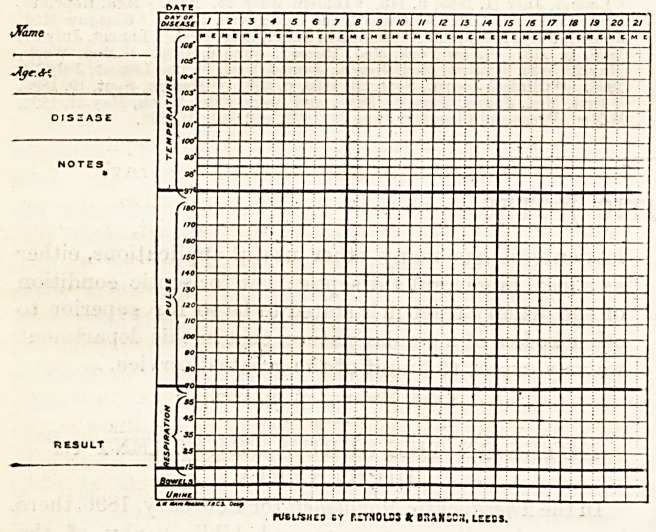# New Appliances and Things Medical

**Published:** 1896-12-26

**Authors:** 


### NEW APPLIANCES AND THINGS MEDICAL.

[We shall be glad to receive, at our Office, 28 & 29, Southampton Street, Strand, London, W.C., from the manufacturers, specimens of all new preparations and appliances which may be brought out from time to time.]

### A NEW CLINICAL CHART.

Messrs. Reynolds and Branson, acting on the suggestion of Professor Mayo Robson, F.R.C.S., have introduced a new clinical chart, of which the accompanying wood-cut is a reduced *fac-simile.* In the ordinary chart the whole attention is directed to the temperature, with very little reference to pulse or respiration. In many cases, however, the pulse has far greater significance than the temperature, this applying

PUBLISHED BY REYNOLDS & BRANSON, LEEDS.

especially to abdominal cases; and it is with a view to being able to compare the daily variations in temperature, pulse, and respiration that the combined chart has been suggested. Daily events can easily be entered—preferably in red ink—and thus the general progress of the case can be judged of at a glance. We understand that this chart was designed by Professor Mayo Robson, who himself uses it.

### CARBOLACENE.

(Messrs. W. and F. Walker, Liverpool.)

We have received a sample of the above preparation, and, after putting it to practical tests, we are prepared to recommend it as a first-class antiseptic cleanser. It is a thick pink fluid which mixes readily with water, and half a pint of it, when diluted with water to about two gallons, constitutes a sufficiently strong solution for ordinary purposes. It may be regarded in the light of an antiseptic liquid soap, and may be used for scrubbing with a brush or rubbing with a flannel; for cleaning woodwork, metals, or paint it is equally efficacious; and, possessing no corrosive or other poisonous action on the skin, it may be used with impunity for washing by manual labour. For disinfecting sick rooms after fever or other infectious diseases it is particularly valuable. It is also well spoken of by those who have used it for destroying vermin in kennels, stables, hen-coops, &c. It has all the antiseptic and cleansing qualities of carbolic soap, without the disadvantages of roughening the skin or possessing poisonous qualities.

### DAVIDSON'S DOUBLE OPTOMETER.

(F. Davidson, 120, Great Portland Street, W.)

This is an instrument which is likely to prove of very great service in examining the condition of the refraction of the eye. The inventor modestly speaks of it as a means of rendering refraction work easy in general practice, but taking the apparatus as a whole, that is both the arrangement of the lenses and that of the card, the saving of time in examining for astigmatism is so considerable that we see no reason why it should not be generally used for subjective examination. So far as the optical part of the apparatus is concerned it consists of a pair of rotating discs, each of which is fitted around its periphery with a number of lenses so arranged that by aid of a few supplementary ones a spherical glass of any number of dioptres, either plus or minus, can be readily brought in front of each eye. The discs are so arranged on a horizontal bar that the distance between the centres of the lenses can be varied to suit the eyes of any patient. Except for the disadvantage of having to use more than one lens to obtain the exact strength of glass required this part of the apparatus is very handy, and the simple rotation of the disc in front of the eye of the patient is in many ways preferable to changing the glasses in the spectacle frame. Much of the advantage, however, of the method advocated by the inventor of this instrument lies in the arrangement of the card on which the test lines are printed, by means of which instead of rotating the lenses for the purpose of investigating astigmatism, the test lines are made to rotate before the eye. By this device the necessity for using cylindrical lenses is done away with, and by the arrangement of the test lines in such form that only two sets at a time can be exposed to view the mental problem put before the patient is much simplified. It is easy to puzzle a patient by asking whether he can see one thing better than another, but there can be no mistake when the only question is whether he can count a row of lines; and if he counts wrongly the error is found out at once. The cards provide means for testing the convergence of the eyes, and certain colour tests are also added, but in regard to these there is nothing special to be said. We were much pleased with the whole arrangement, and think it is likely to prove of considerable utility.

## Figures and Tables

**Figure f1:**